# Generation of a *Tph2* Conditional Knockout Mouse Line for Time- and Tissue-Specific Depletion of Brain Serotonin

**DOI:** 10.1371/journal.pone.0136422

**Published:** 2015-08-20

**Authors:** Barbara Pelosi, Marta Pratelli, Sara Migliarini, Giulia Pacini, Massimo Pasqualetti

**Affiliations:** 1 Department of Biology, Unit of Cell and Developmental Biology, University of Pisa, S.S.12 Abetone e Brennero 4, 56127, Pisa, Italy; 2 Center for Neuroscience and Cognitive Systems@UniTn, Istituto Italiano di Tecnologia, Via Bettini 31, 38068, Rovereto (TN), Italy; Max-Delbrück Center for Molecular Medicine (MDC), GERMANY

## Abstract

Serotonin has been gaining increasing attention during the last two decades due to the dual function of this monoamine as key regulator during critical developmental events and as neurotransmitter. Importantly, unbalanced serotonergic levels during critical temporal phases might contribute to the onset of neuropsychiatric disorders, such as schizophrenia and autism. Despite increasing evidences from both animal models and human genetic studies have underpinned the importance of serotonin homeostasis maintenance during central nervous system development and adulthood, the precise role of this molecule in time-specific activities is only beginning to be elucidated. Serotonin synthesis is a 2-step process, the first step of which is mediated by the rate-limiting activity of Tph enzymes, belonging to the family of aromatic amino acid hydroxylases and existing in two isoforms, Tph1 and Tph2, responsible for the production of peripheral and brain serotonin, respectively. In the present study, we generated and validated a conditional knockout mouse line, *Tph2* ^*flox/flox*^, in which brain serotonin can be effectively ablated with time specificity. We demonstrated that the Cre-mediated excision of the third exon of *Tph2* gene results in the production of a *Tph2* ^*null*^ allele in which we observed the near-complete loss of brain serotonin, as well as the growth defects and perinatal lethality observed in serotonin conventional knockouts. We also revealed that in mice harbouring the *Tph2* ^*null*^ allele, but not in wild-types, two distinct *Tph2* mRNA isoforms are present, namely *Tph2Δ3* and *Tph2Δ3Δ4*, with the latter showing an in-frame deletion of amino acids 84–178 and coding a protein that could potentially retain non-negligible enzymatic activity. As we could not detect *Tph1* expression in the *raphe*, we made the hypothesis that the *Tph2Δ3Δ4* isoform can be at the origin of the residual, sub-threshold amount of serotonin detected in the brain of *Tph2* ^*null/null*^ mice. Finally, we set up a tamoxifen administration protocol that allows an efficient, time-specific inactivation of brain serotonin synthesis. On the whole, we generated a suitable genetic tool to investigate how serotonin depletion impacts on time-specific events during central nervous system development and adulthood life.

## Introduction

The biosynthesis of the monoaminergic neurotransmitter serotonin (5-hydroxytryptamine, 5-HT) has as its first and rate-limiting step the conversion of the aminoacid L-tryptophan into 5-hydroxytryptophan (5-HTP), catalysed by the enzyme tryptophan hydroxylase (Tph). In mammals, it has been demonstrated the existence of two Tph isoforms, codified by either *Tph1* or *Tph2* gene [1,2]. Together with phenylalanine hydroxylase (PAH) and tyrosine hydroxylase (TH), Tph belongs to the family of pterin-dependent aromatic amino acid hydroxylases (AAAHs), having BH_4_ and O_2_ as co-substrates and Fe^2+^ as cofactor. Crystallographic and mutagenesis studies have shown that AAAHs are characterized by three main functional regions: a regulatory N-terminal region, a catalytic domain, which contains the cofactor and substrate binding sites, and the C-terminal leucine zipper tetramerization domain [3–5]. Despite Tphs have a protein sequence homology of 71%, Tph2 has 44 additional aminoacidic residues at its N-terminus, which are not present in Tph1 [1]. Tph1 and Tph2 enzymes differ also in terms of their spatial distribution. Tph1 is predominantly expressed in the entherocromaffin cells of the gut, accounting for the main source of circulating 5-HT, and in the pineal gland, where serotonin is the precursor of melatonin [6,7]. Tph2 is expressed in the myenteric plexus [8] and in the serotonergic neurons of the *raphe* nuclei localized in the brainstem, where it is responsible for the synthesis of central serotonin [2,9,10].

In mice, serotonergic neurons develop around embryonic day 11 and in the adult brain collectively form the *raphe* nuclei, which provide a dense innervation to the whole rostral brain as well as to the spinal cord [9,11–13]. In line with such a broad distribution of serotonergic innervation, serotonin is known to be implicated in the modulation of numerous physiological processes, including the control of sleep, appetite, sexual behaviour, mood and cognition [14–19]. In the last two decades, the growing interest in serotonergic system has resulted in the generation of multiple mouse models in which serotonergic signalling has been perturbed. Notably, mouse lines targeting genes codifying for serotonin receptors [20–23], for enzymes involved in 5-HT synthesis as Tph1 and Tph2 [6,9,24–26], in its metabolism as monoaminoxidase A (MAOA) [27] or in its re-uptake as serotonin transporter (SERT) [28,29], have been generated. Such genetic tools, together with the detection of maternal and placental sources of serotonin [30,31] have suggested that serotonin can behave as a key regulator of specific events during development, contributing to cell proliferation, migration, neuronal differentiation and brain circuitry formation [9,29,32–38]. In line with that, post-mortem studies on human samples as well as analysis of mouse tools have provided insights on the role of 5-HT neurotransmission impairment on the onset of human neuropsychiatric disorders thought to have a developmental origin, like schizophrenia, autism, affective disorders, anxiety, depression and mental retardation [16,39–46].

In this regard, during the last few years different *Tph2* knockout mouse lines, in which the synthesis of serotonin is selectively abrogated in the brain, have been generated by distinct lab groups [9,24–26,47]. Despite these mouse lines have provided valuable insights to understand the role of serotonin, the inactivation of *Tph2* and consequent depletion of serotonin *ab origine* may lead to compensatory responses during development, which would mask the precise involvement of serotonin at postnatal stages or in adults, as often seen in conventional knockout mice [48,49]. The spatio-temporal control of *Tph2* gene inactivation through the generation of conditional knockout alleles can overcome these limits. To our knowledge, three distinct conditional *Tph2* knockout alleles have been generated [50–52]. Among these lines, only one has been used as an inducible *Tph2* knockout, though upon Cre-mediated somatic recombination numerous serotonergic neurons were still present in the *raphe* of these animals [51].

Here we report the generation of a new conditional allele of the *Tph2* gene, namely *Tph2* ^*flox*^, in which the third exon of *Tph2* gene is flanked by *loxP* sites. Our data demonstrated that the presence of the *loxP* sites did not impact on the normal serotonin synthesis and that, following a CMV-driven Cre-mediated excision of the third exon, a *Tph2* null allele (*Tph2* ^*null*^) was generated. *Tph2* ^*null/null*^ mice displayed early postnatal mortality and growth defects recapitulating the phenotypic features observed in *Tph2* conventional knockouts. Finally, we describe the set up of a tamoxifen treatment to deplete brain serotonin in a time-controlled manner following Cre-mediated somatic recombination.

## Materials and Methods

### Animals

Mice were housed in standard Plexiglas cages with food and water *ad libitum* and maintained at a constant temperature of 22 +/- 1°C on an artificial 12/12 h light/dark cycle. Experimental protocols were conducted in accordance with the Ethic Committee of the University of Pisa and approved by the Veterinary Department of the Italian Ministry of Health.

For the experiments mice on C57BL/6 genetic background were used.

Mice subjected to pharmacological treatments, as well as respective controls, were housed in single cages.

### Generation of the *Tph2* ^*flox*^ Knockin Mouse Line

To generate *Tph2* ^*flox*^ knockin allele we took advantage of a homologous recombination strategy carried out in mouse ES cells. For *Tph2* ^*flox(Neo)*^ targeting vector preparation, a genomic DNA fragment comprising 3486 bp upstream the third exon and 3456 bp downstream the fourth exon of *Tph2* gene was isolated using Nhel and Eco47III restriction enzymes and cloned in a pBluescript KS plasmid. A selection marker cassette cloned 5’ to a *loxP* site and flanked by two *FRT* sites for Flp mediated excision, was inserted in the NcoI unique restriction site upstream *Tph2* third exon. A second *loxP* site was inserted, with the same orientation, 3’ to the *Tph2* third exon in the HpaI restriction site, resulting in a 574 bp floxed region. The resulting *Tph2* ^*flox*^ targeting vector was then SalI linearized and electroporated into E14Tg2a.4 embryonic stem cells (ES). Neomycin-resistant cells were screened using Southern blot and one clone carrying the desired integration in the *Tph2 locus* was identified. Recombinant ES cells were microinjected into C57BL/6 host blastocysts, which were subsequently implanted into pseudopregnant females to generate chimeric mice. Two chimeric males showed germline transmission of the *Tph2* ^*flox(Neo)*^ allele. To excise the *PGK-Neo* resistance cassette, *Tph2* ^*flox(Neo)/+*^ heterozygous mice were crossed with the *ACTB*::*FLPe* deleter [53], generating the *Tph2* ^*flox*^ mouse line. Animals on mixed genetic background were then backcrossed with C57BL/6 mice for at least nine generations before being used for experiments.

Two transgenic mouse lines expressing Cre recombinase were used to achieve Cre-mediated somatic recombination in the *Tph2* ^*flox*^ allele, thus leading to *Tph2* third exon excision. A CMV-Cre deleter mouse line, in which Cre expression is driven by the constitutive human cytomegalovirus minimal promoter [54], was used to achieve the ubiquitous deletion of *Tph2* third exon. In order to obtain a time-inducible *Tph2* knockout, we took advantage of the *CMV-CreER* ^*T*^ mouse line, expressing a fusion protein between the Cre recombinase and the mutated ligand-binding domain of the human estrogen receptor under the control of an ubiquitous promoter. Cre activity in this mouse line can be conditionally induced through administration of Tamoxifen (TM; [55])

### Genotyping

To identify the presence of *Tph2* ^*flox*^ and *Tph2* ^*null*^ allele, mice were routinely genotyped using standard PCR conditions. The following primers: forward 5’-TGGTCTACAGAGTGAGTTCCAAGA-3’ and reverse 5’-TCTGGAAGAGTGTTGACTGTGTTAG-3’ were used to distinguish between the *Tph2* wild-type and the *Tph2* ^*flox*^ allele, from which, respectively, a 433 bp and a 501 bp fragment were amplified. Using the same forward primer in combination with the reverse primer 5’-CACGGCACATCCTCGAGATC-3’ the wild-type (849 bp) and the *Tph2* ^*null*^ allele (343 bp) were discriminated.

### Immunohistochemistry and *In Situ* Hybridization Analysis

Immunohistochemistry and *in situ* hybridization analysis were carried out following previously described standardized protocols [9,56].

Briefly, to collect tissues for immunohistochemical analysis, both pups and adult mice were anaesthetized by intraperitoneal injection of Avertin (1.25% solution of 2, 2, 2-Tribromoethanol, Sigma-Aldrich) at 0.02 ml/g body weight and perfused transcardially with 4% paraformaldehyde (PFA). Brains were dissected out and post-fixed overnight (o/n) at 4°C in 4% PFA. Coronal 50μm thick sections were obtained with a vibratome (Leica Microsystems). For immunostaining, free-floating sections were incubated o/n at 4°C with rabbit anti-5-HT primary antibody (Sigma-Aldrich) diluted 1:500 in a solution of phosphate buffered saline (PBS) containing 5% horse heat-inactivated serum and 0.5% Triton X-100. After one day washing with 0.5% Triton X-100 in PBS solution, sections were incubated o/n with a fluorescent-conjugated Rhodamine Red-X goat anti-rabbit secondary antibody (Molecular Probes) in a dilution of 1:500.

Fluorescent images were acquired using a 10x or 40x objective mounted on a Leica TCS SP8 confocal microscope.

For radioactive *in situ* hybridization, fresh brains embedded in TISSUE Tek (Sakura) were frozen in dry ice and stored at -80°C. *In situ* hybridization was performed using ^35^S-labelled *Tph1* antisense RNA probes (PG4TH plasmid, American Type Culture Collection, University Boulevard, Manassas, USA) on 14 μm thick cryostat sections, later exposed to Biomax MR X-ray films (Kodak) for one to five days. Brightfield pictures were acquired using a MacroFluo microscope (Leica) equipped with a DS-SMc digital camera (Nikon).

### Total RNA Extraction and RT-PCR Analysis

For RNA extraction adult wild-type, *Tph2* ^*null/null*^ and tamoxifen-treated *Tph2* ^*flox/null*^::*CMV-CreER* ^*T*^ mice were anesthetized with Avertin and euthanized by decapitation. Tamoxifen treated mice were sacrificed seven days after the last tamoxifen injection. Brains were rapidly dissected out and total RNA was extracted from the *raphe* using TRIzol reagent (Life Technologies) according to the manufacturer's instructions. Reverse transcriptase reactions were performed on 500ng of RNA using oligo(dT) primers (Promega) and ImProm-II reverse transcriptase (Promega) in parallel with a control reaction without reverse transcriptase.

The amount of cDNA was normalized based on the expression of the housekeeping gene *β-actin*. Primer sequences were as follows: *β-actin* forward: 5’-AGGTCATCACTATTGGCAACGA-3’, reverse: 5’-CCGATCCACACAGAGTACTTG-3’; *Tph1* forward: 5’-CATTAGAAGTATGTCCACGGGC-3’, reverse: 5’-ACTCTCCCTCTTTCGGAGGA-3’; *Tph2* forward: 5’-CAGTTCCTCCTTCATCTCTGC-3’, reverse: 5’-CGCTTTTCTTGTCCTCGC-3’.

### Analysis of *Tph2* ^*null*^ Transcript

Total *raphe* cDNA was amplified in 10 parallel PCR reactions. In each reaction a common forward primer (Fw1) designed within *Tph2* exon 1 was used in combination with a reverse primer specifically designed within each distinct *Tph2* exon, from exon 2 to exon 11 (namely Rev2 to Rev11). Exon 1 forward primer Fw1: 5’-CAGTTCCTCCTTCATCTCTGC-3’; Exon 2 reverse primer Rev2: 5’-CGCTTTTCTTGTCCTCGC-3’; Exon 3 reverse primer Rev3 5’-CTCATTGAATTCCGTTTTGCCAC-3’; Exon 4 reverse primer Rev4: 5'-CGGTGAGAGCATCTGTCTAAC-3'; Exon 5 reverse primer Rev5: 5'-CTCTGTCGATAGACATTGTCC-3'; Exon 6 reverse primer Rev6: 5'-GAGAGCTCCCGGAACACAAC-3'; Exon 7 reverse primer Rev7: 5'-TCACTGTGAAGCCAGATCGC-3'; Exon 8 reverse primer Rev8: 5'-AGCAAACTTGGGATCCGCAAGC-3'; Exon 9 reverse primer Rev9: 5'-TTGCAAAGGCCGAACTCGATT-3'; Exon 10 reverse primer Rev10: 5'-ATTTCACACACGCCTTGTCGG-3'; Exon 11 reverse primer Rev11: 5'-GGTCCTGCACCACATTCTCA-3'. PCR reactions were performed with GoTaq (Promega) DNA polymerase, using the following thermal cycling conditions: denaturation at 94°C for 25 seconds, annealing at 60°C for 30 seconds, elongation at 72°C for one minute, for a total of 35 cycles.

Amplicons obtained using Fw1 and Rev5 primers were gel-extracted using MinElute PCR purification kit (Qiagen) and sequenced.

### Drug Treatment

Following a previously established protocol [57] tamoxifen (Sigma) was dissolved in sunflower oil/ethanol 9:1 at the concentration of 10mg/ml. Animals at postnatal day 60 (P60) were injected intraperitoneally once a day for 5 consecutive days with 75mg/kg body weight with tamoxifen or oil/ethanol (9:1) alone (vehicle; [58]).

### Data and Statistical Analysis

For serotonin positive cell counting, immunohistochemistry experiments were performed on animals sacrificed 1, 3, 7, 30, 60 or 90 days after the end of tamoxifen treatment (three to six animals were used for each time point). Serotonin positive cells showing a labelling clearly above the background level were counted using the Image-J software on three representative sections of the median and dorsal *raphe* nuclei for each animal.

Survival analysis was performed using Kaplan–Meier method followed by a Log-rank test. Growth rate and cell count analyses were performed using analysis of variance (ANOVA) combined with Tukey’s or Fisher’s post hoc test. Data are expressed as mean +/- SEM.

## Results

### Generation of the *Tph2* ^*flox*^ Allele

**Fig 1:**
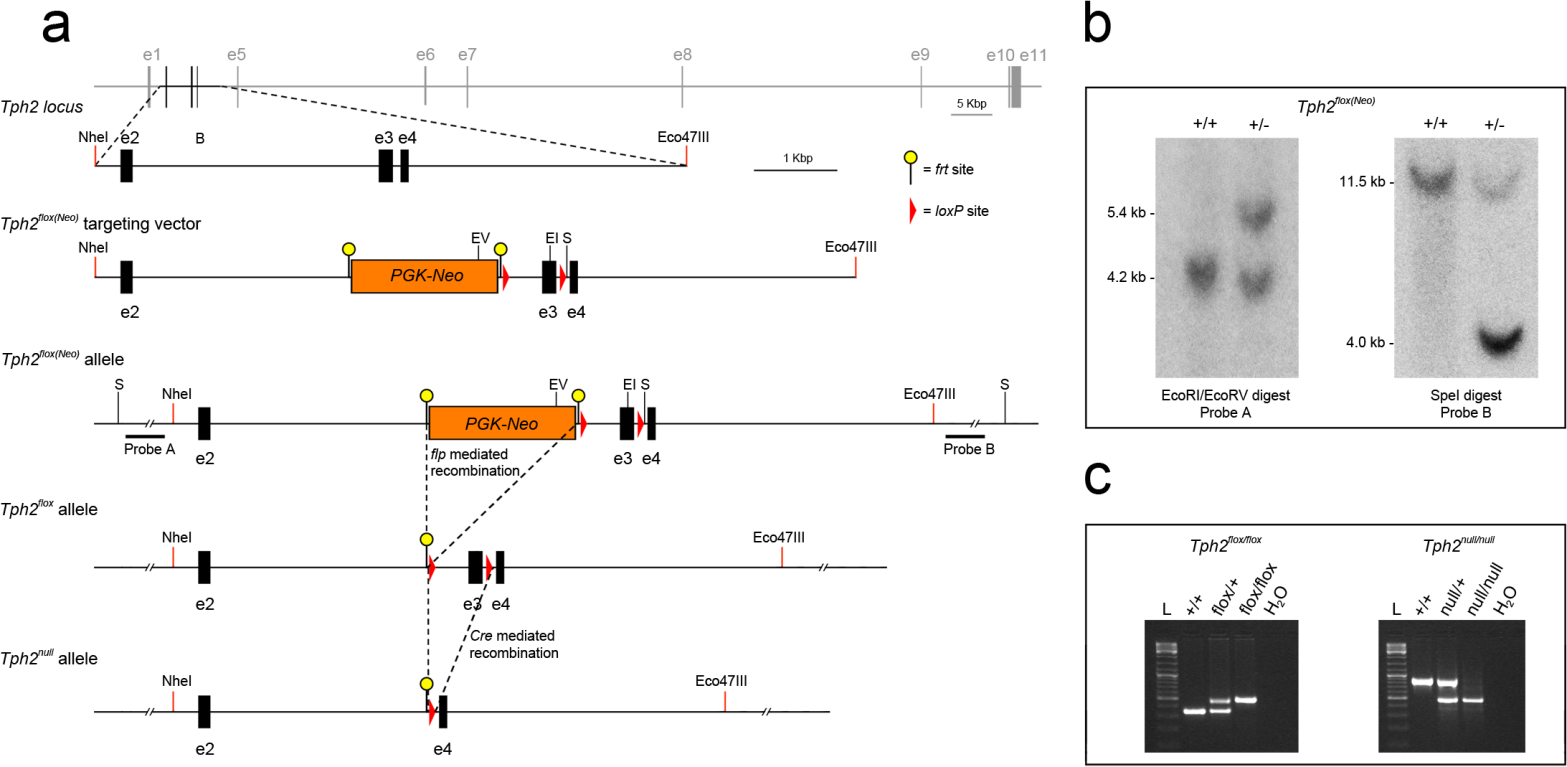
Generation of the *Tph2* ^*flox*^ and *Tph2* ^*null*^ alleles. (**a**) Diagram showing the targeting strategy used to generate the *Tph2* ^*flox(Neo)*^ allele and the derivation of both *Tph2* ^*flox*^ and *Tph2* ^*null*^ alleles after the removal of the *PGK-neo* cassette via flp mediated recombination and the removal of the third exon of *Tph2* gene after Cre mediated somatic recombination, respectively. Schematic representation of wild-type *Tph2* genomic locus, *Tph2* ^*flox(Neo)*^ targeting vector, *Tph2* ^*flox(Neo)*^, *Tph2* ^*flox*^ and *Tph2* ^*null*^ alleles are reported. (**b**) Southern blot analysis confirming the correct integration of *Tph2* ^*flox(Neo)*^ targeting vector within the genome of ES cells via homologous recombination. Genomic DNA was digested with EcoRI and EcoRV, or SpeI restriction enzymes and hybridized with a probe external to the left homology arm (probe A) or to the right homology arm (probe B), respectively. Probe A and probe B are indicated in **a**. (**c**) PCR genotyping of mice using tail biopsy allowing to discriminate wild-type (*Tph2* ^*+/+*^), *Tph2* ^*flox/+*^, *Tph2* ^*flox/flox*^, as well as *Tph2* ^*null/+*^ and *Tph2* ^*null/null*^ animals. EV: EcoRV; EI: EcoRI; S: SpeI; wt: wild-type; L: ladder.

In order to generate a *Tph2* conditional knockout mouse line (*Tph2* ^*flox/flox*^) we designed a targeting vector, *Tph2* ^*flox(Neo)*^, in which 5-HT synthesis can be abrogated in a tissue-specific and time-controlled manner. *Tph2* gene spans over 100 Kb and contains 11 exons, thus preventing the possibility to flox the entire gene (Fig 1A). We decided to introduce *loxP* sites into introns flanking exon 3 as it contains a number of nucleotides (184 bp in length codifying for aminoacids 84–145) that is not an exact multiple of 3, so that a Cre mediated recombination event results in a frameshift leading to an early stop codon (i.e. nucleotides 43–45 of exon 4), likely producing a *Tph2* null allele. A *PGK-Neo* selection cassette flanked by *FRT* sites was inserted adjacent of *loxP* site within intron 2 as well (Fig 1A).

The *Tph2* ^*flox(Neo)*^ targeting vector was introduced into mouse embryonic stem cells (mES) by electroporation and recombinant clones in which a homologous recombination event had occurred were identified by Southern Blot analysis (Fig 1B). Recombinant mES cells were then microinjected into C57BL/6 host blastocysts to generate chimeric mice, which were then tested for germline transmission. Once obtained, *Tph2* ^flox(Neo)/+^ heterozygous mice were crossed with the *ACTB*::*FLPe* deleter mouse line in order to remove the *PGK-Neo* cassette, which has been reported to interfere with gene expression (Fig 1A; [9,59]). *Tph2* ^flox/+^ mice were mated with C57BL/6 wild-type mice for nine generations before being considered on a pure C57BL/6 background, and subsequently intercrossed to obtain homozygous *Tph2* ^flox/flox^ mice. All the genotypes were confirmed by PCR analysis (Fig 1C). *Tph2* ^flox/flox^ mice were born at normal Mendelian ratio and were viable without displaying any obvious defect as compared to wild-type littermates. Adult *Tph2* ^flox/flox^ females generated normal-sized litters and showed normal maternal care behaviour (not shown).

### *Tph2* ^*null/null*^ Mice Show 5-HT Depletion-Like Features

**Fig 2:**
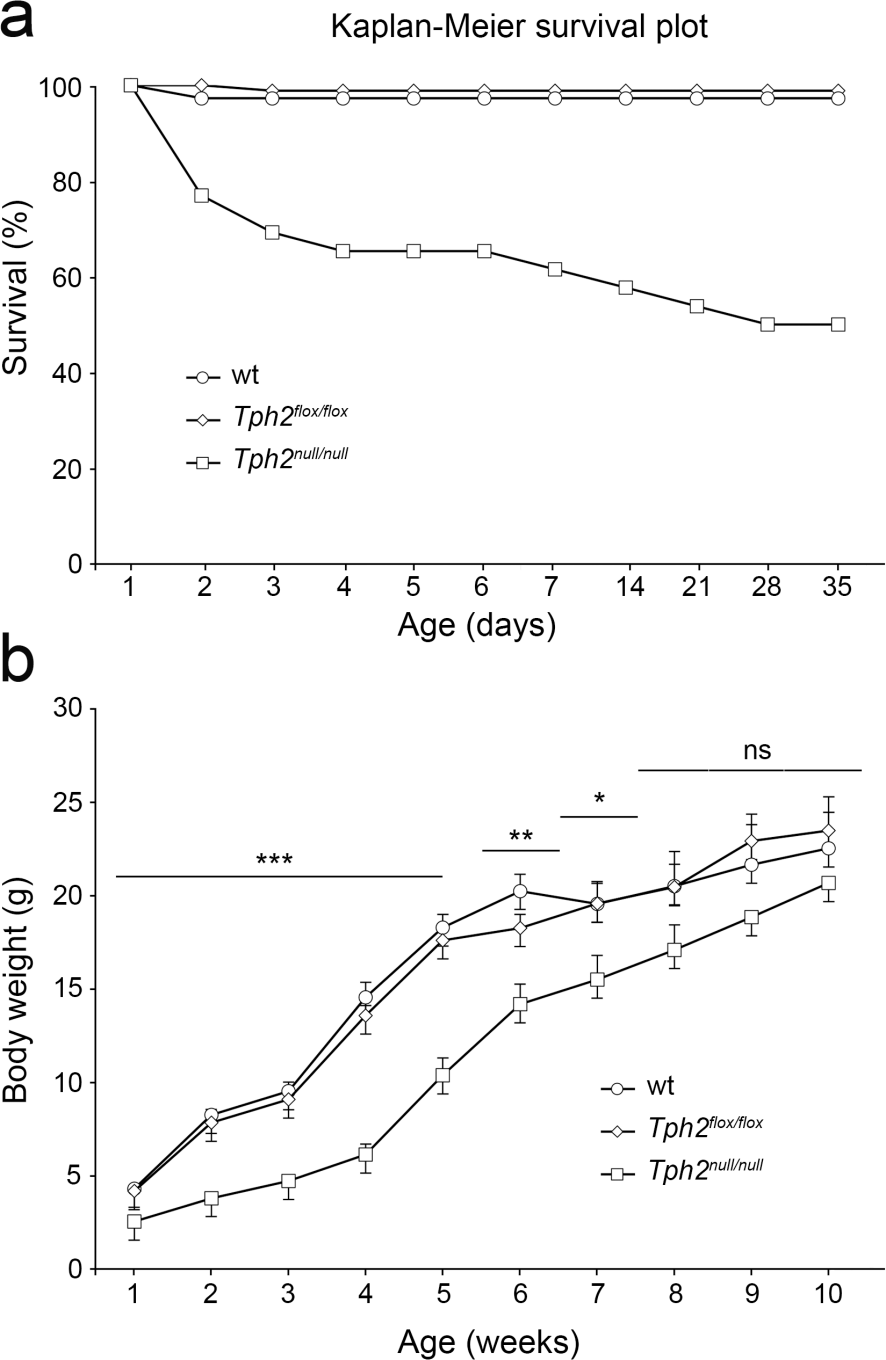
Postnatal lethality and impaired growth rate in *Tph2* ^*null/null*^ mice. (**a**) Kaplan–Meier survival curve shows the survival rate of *Tph2* ^*null/null*^ pups (n = 61) within the first 35 postnatal days in comparison with wild-type (n = 64) and *Tph2* ^*flox/flox*^ (n = 70) siblings. (**b**) Animals of the three genotypes were weighted weekly for the first 10 postnatal weeks. The graph shows that *Tph2* ^*null/null*^ mice display a significant weight reduction compared to wild-type and *Tph2* ^*flox/flox*^ during the first 7 weeks after birth. Data are presented as mean ± SEM. ***p<0,001, **p<0,01, *p<0,05, ns: not significant.

It has been previously shown that *Tph2* gene inactivation results in the ablation of serotonin content within the brain, together with a striking impairment in growth rate and in viability of *Tph2*-deficient mice [9,24,44]. In order to assess the consequences of Cre–mediated excision of the third exon of *Tph2* gene on Tph2 activity and mouse phenotype, we intercrossed *Tph2* ^flox/+^ mice with CMV-Cre deleter mice [54], to obtain *Tph2* ^*null/+*^ mice in which exon 3 of the *Tph2* gene was constitutively missing on the recombinant allele (Fig 1A). *Tph2* ^*null/null*^ mice were then derived by intercrossing *Tph2* ^*null/+*^ animals and genotypes were confirmed by PCR (Fig 1C). A striking postnatal lethality in *Tph2* ^*null/null*^ pups was already evident within few days after birth (Fig 2A). Indeed, only 61% of mutant mice survived the first week after birth, as compared with 97% and 98% of wild-type littermates or *Tph2* ^flox/flox^ mice, respectively. Lethality was scored up to the end of the fifth week of age when in *Tph2* ^*null/null*^ mice it reached the 50%, whereas it did not significantly change in *Tph2* ^flox/flox^ or wild-type controls (Fig 2A). Further, analysis of the growth rate highlighted a statistically significant deficit in weight gain of *Tph2* ^*null/null*^ mice within the first seven weeks after birth, as compared to both wild-type or *Tph2* ^flox/flox^ mice (Fig 2B). Starting from the second month of age, *Tph2* ^*null/null*^ mice progressively recovered their growth delay and after the third month of age the difference in body weight was not obvious anymore. Once reached adulthood, *Tph2* ^*null/null*^ animals were fertile and lived a normal life span.

**Fig 3:**
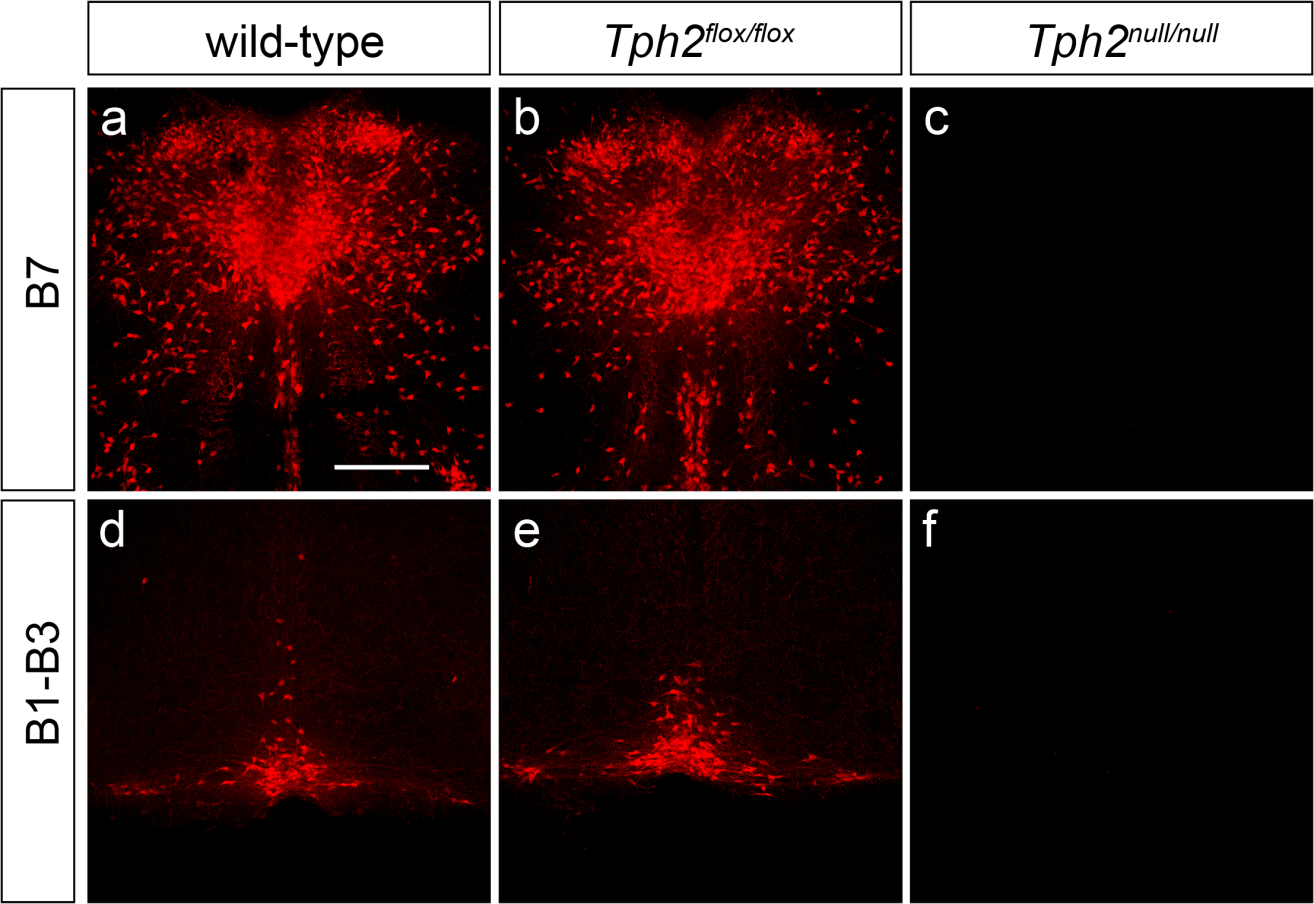
*Tph2* ^*null/null*^ mice are depleted of brain serotonin. Representative confocal images of P0 wild-type (**a**, **d**), *Tph2* ^*flox/flox*^ (**b**, **e**) and *Tph2* ^*null/null*^ (**c**, **f**) brain coronal sections, showing the distribution of 5-HT immunoreactive neurons within B7 (**a**-**c**) and B1-B3 (**d**-**f**) *raphe* nuclei. While no differences are visible in the distribution of serotonin immunoractive neurons between wild-type and *Tph2* ^*flox/flox*^ pups (**a**-**b, d**-**e**), the brain of *Tph2* ^*null/null*^ pups appear to be devoid of 5-HT throughout its anterior-posterior axis (**c**, **f**). Scale bar: 400 μm.

As both the growth defect and the postnatal mortality seen in the *Tph2* ^*null/null*^ mice were in line with what observed in serotonin-deficient *Tph2* knockouts [9,24], we next wanted to determine whether the defects observed in *Tph2* ^*null/null*^ mice were resulting from a depletion of brain serotonin content as a consequence of the excision of the third exon of *Tph2*. We thus performed immunohistochemistry analysis on coronal sections of *Tph2* ^*null/null*^, *Tph2* ^flox/flox^ and wild-type mouse brains throughout the antero-posterior extent of serotonergic *raphe* nuclei, both at birth and in adult animals (Fig 3 and S1 Fig). At P0, serotonin immunoreactive neurons were present in the *raphe* nuclei of *Tph2* ^flox/flox^ mice and normally distributed as compared to wild-type controls (Fig 3A, 3B, 3D and 3E). This evidence, together with the absence of any noticeable developmental or behavioural phenotype, demonstrated that *loxP* sites flanking the third exon of *Tph2* gene did not interfere with proper regulation of the *Tph2 locus*. Conversely, anti-serotonin immunostaining was not detectable in *Tph2* ^*null/null*^ animals at either rostral (B7) or caudal (B1-B3) *raphe* nuclei (Fig 3C and 3F). Results from immunohistochemical analysis performed at P60 confirmed that the serotonergic system appearance was undistinguishable between *Tph2* ^flox/flox^ and wild-type animals, along with undetectable levels of serotonin immunoreactivity in the *raphe* of *Tph2* ^*null/null*^ mice, as observed at earlier stages (S1A–S1C and S1D–S1F Fig). In spite of that, closer observation at higher sensitivity achieved combining the use of confocal laser scanning microscope and photomultiplier tubes or artificial gain allowed to detect the presence of anti-serotonin immunofluorescence in few scattered neurons throughout the *raphe* nuclei of *Tph2* ^*null/null*^ mice, suggesting the existence of a remnant of 5-HT (S1C’ and S1F’ Fig). The same detection procedure was then applied to *raphe* sections of *Tph2*::*eGFP*-/- knockin mice, in which EGFP replaces the first *Tph2* exon resulting in complete Tph2 enzymatic activity abrogation [9]. Conversely to what observed in *Tph2* ^*null/null*^ mice, the analysis on *Tph2*::*eGFP*-/- specimens failed to show any serotonergic neuron-like labelling beyond the background fluorescence, thus confirming the specificity of the immunoreactivity observed in *Tph2* ^*null/null*^ mice (S1C” and S1F” Fig).

Therefore, despite serotonin depletion in *Tph2* ^*null/null*^ mice was not complete, these data demonstrated that Cre-mediated excision of the third exon of *Tph2* is sufficient to promote phenotypic features analogous to those observed in *Tph2* knockout mouse lines [9,24].

### A *Tph2* Splicing Variant with Restored Open Reading Frame Is Produced in *Tph2* ^*null/null*^ Mice

The presence of a remnant of serotonergic immunostaining in the *raphe* of *Tph2* ^*null/null*^ mice, although barely detectable, prompted us to investigate its possible origin. It is known that Tph exists in two isoforms, namely Tph2, which is responsible for serotonin synthesis in the *raphe* nuclei, and Tph1, which is expressed in enterochromaffin cells of the gastrointestinal tract [1], as well as in the pineal gland and in the placenta [30]. Few reports also argued for a transient *Tph1* expression in the brain, but this hypothesis is still controversial [10,60]. We wanted to assess whether the presence of serotonin in the *raphe* of *Tph2* ^*null/null*^ mice could have a Tph1 origin and to this aim we first checked for potential *Tph1* expression by means of both *in situ* hybridization (ISH) and RT-PCR on adult *raphe*. Results ruled out the presence of *Tph1* in the serotonergic neurons of *Tph2* ^*null/null*^ mice, excluding a *Tph1*-mediated compensatory mechanism for the synthesis of serotonin in the *raphe* nuclei of *Tph2* ^*null/null*^ mice (S2 Fig). Subsequently, although it is largely accepted that serotonin could not reach the brain from the circulating blood via the blood–brain barrier (BBB), we wanted to exclude that the expression of the serotonin transporter (SERT) in both the vascular endothelial cells of the BBB [61,62] and in serotonergic neurons [63] could be responsible for the presence of serotonin in the *raphe* of *Tph2* ^*null/null*^ mice. To test this hypothesis, we blocked SERT in *Tph2* ^*null/null*^ adult mice through a two-week administration of fluoxetine, a specific serotonin reuptake inhibitor. Results revealed no differences in serotonergic immunoreactivity between fluoxetine-treated versus control mice (data not shown), confirming that the origin of the residual serotonin was likely independent from Tph1 enzymatic activity.

**Fig 4:**
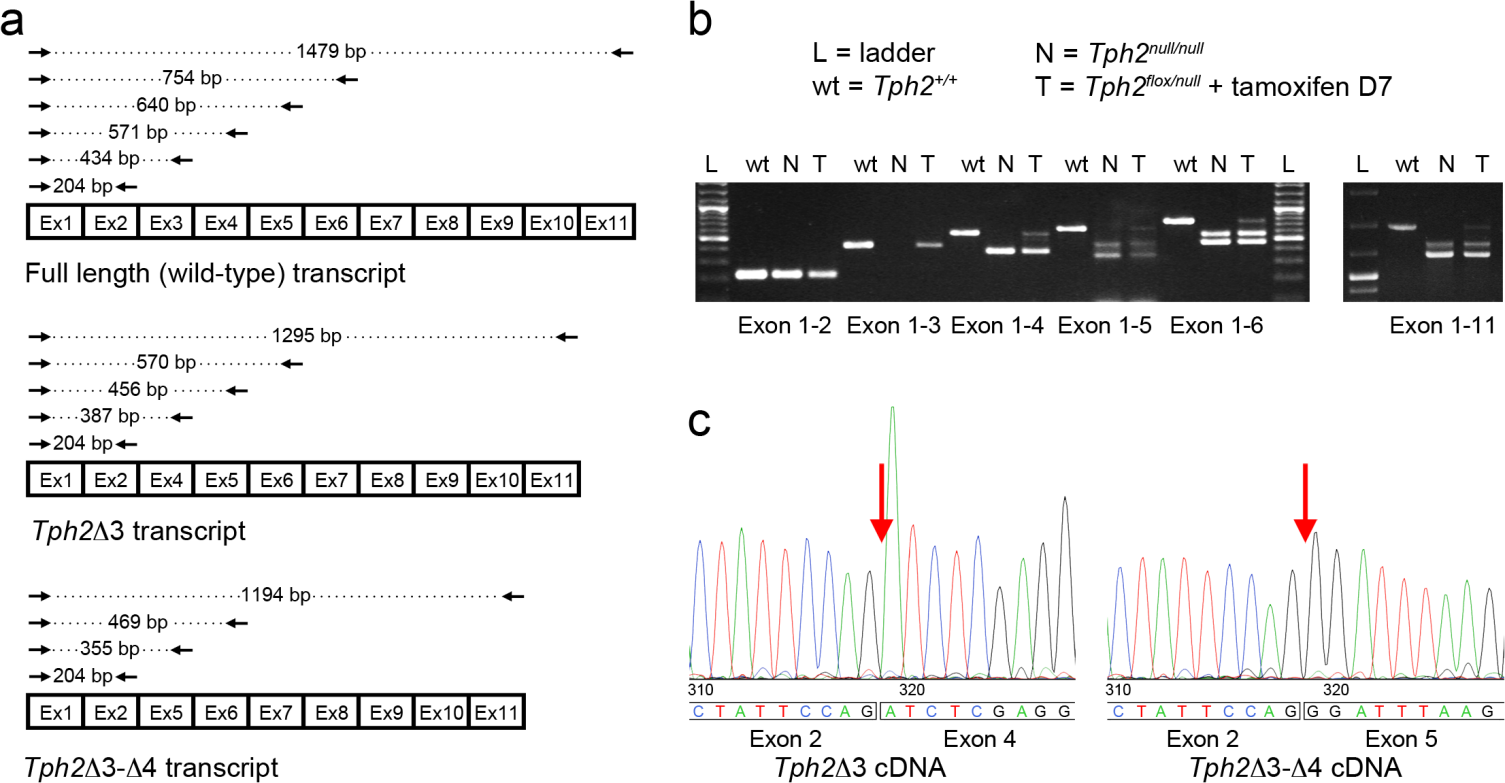
Characterization of *Tph2* ^*null*^ transcripts. (**a**) Schematic representation of RT-PCR products obtained from *Tph2* ^*+/+*^, *Tph2Δ3* and *Tph2Δ3Δ4* transcripts using the following primer sets: Fw1 –Rev2, Fw1 –Rev 3, Fw1 –Rev 4, Fw1 –Rev5, Fw1 –Rev6 and Fw1 –Rev11. (**b**) Agarose 1.5% gel showing the RT-PCR products obtained from *Tph2* ^*+/+*^, *Tph2* ^*null/null*^ and tamoxifen-treated *Tph2* ^*flox/null*^::*CMV-CreER* ^*T*^ cDNA using the primer sets described in (**a**). Two distinct PCR products are obtained using Fw1 –Rev 4 (exon1-4), Fw1 –Rev 5 (exon1-5), Fw1 –Rev 6 (exon1-6), and the Fw1 –Rev 11 (exon1-11) primer sets from cDNA of *Tph2* ^*null/null*^ and the tamoxifen treated *Tph2* ^*flox/null*^::*CMV-CreER* ^*T*^ mice. An additional amplicon corresponding in size to the wild-type PCR product is amplified from *Tph2* ^*flox/null*^::*CMV-CreER* ^*T*^ cDNA. (**c**) Electropherograms showing the nucleotide sequence obtained sequencing the two distinct amplicons obtained from *Tph2* ^*null/null*^ cDNA using Fw1—Rev5 primers. Sequencing analysis shows that the second exon of the *Tph2* transcript is joined to the fourth exon in the *Tph2Δ3* cDNA, while it is directly connected to the fifth exon in the *Tph2Δ3Δ4* transcript. L: ladder; N: cDNA from the *raphe* of a *Tph2* ^*null/null*^ mice; T: cDNA from the *raphe* of a *Tph2* ^*flox/null*^::*CMV-CreER* ^*T*^ tamoxifen-treated mouse sacrificed seven days after the end of the treatment.

We finally considered that in the *raphe* of *Tph2* ^*null/null*^ mice Tph2 might maintain a functional activity even upon excision of the third exon. We therefore examined *Tph2* transcript by *in silico* analysis and results revealed the possibility that alternative splicing events could restore the open reading frame and produce a shorter but potentially functional low-active form of Tph2 enzyme. To further investigate this possibility, we extracted total RNA from the *raphe* of wild-type and *Tph2* ^*null/null*^ mice, and RT-PCR analysis was performed using a universal forward primer (Fw1) designed within the first exon of the *Tph2* gene, in combination with a specific reverse primer in turn positioned in each other exon (namely Rev2 to Rev11; Fig 4A). The amplified fragments from wild-type cDNA obtained with each combination of Fw1 and Rev (2–11) primers showed a ladder pattern when analysed by gel electrophoresis, with a specific single band of expected size for each of the 10 *Tph2* Rev primers (Fig 4B). RT-PCR results from *Tph2* ^*null/null*^ mice cDNA showed a band of identical size as from wild-type cDNA when performed with primers Fw1—Rev2, while no amplification was detected with primers Fw1—Rev3 because of the genetic removal of the third exon and, as expected, a 184 bp shorter amplification product was obtained with Fw1—Rev4 primers (Fig 4B). Strikingly, when PCR was performed using Fw1 in combination with any of the reverse primers from Rev5 to Rev11, in addition to the band of the predicted size, a shorter amplification product was also obtained with each primer set (Fig 4B). Analysis of these results suggested that the additional band could derive from a splicing variant of the *Tph2* mRNA, lacking both the third and the fourth exon. To validate this hypothesis, we gel extracted and sequenced the two distinct amplicons obtained with Fw1—Rev5 amplification, in order to examine the nucleotide sequence at the junction between the exons. Results showed that the longest fragment amplified with Fw1—Rev5 primers corresponded to the mRNA isoform lacking the third exon of *Tph2* gene (*Tph2Δ3*; Fig 4C), whereas the shortest band identified a distinct mRNA isoform, in which the second *Tph2* exon was directly connected to the fifth exon, thus lacking both the third and the fourth exon (*Tph2Δ3Δ4*; Fig 4C). Importantly, this new mRNA splicing isoform was characterized by a number of nucleotides that is evenly divisible by three. Thus, in the *Tph2Δ3Δ4* isoform, unlike in *Tph2Δ3* transcript, the correct reading frame was restored and was predicted to encode a protein with an in-frame deletion of amino acids 84–178, which could potentially retain non-negligible enzymatic activity.

### Time-Specific Removal of *Tph2* Third Exon Results in Brain Serotonin Depletion

**Fig 5:**
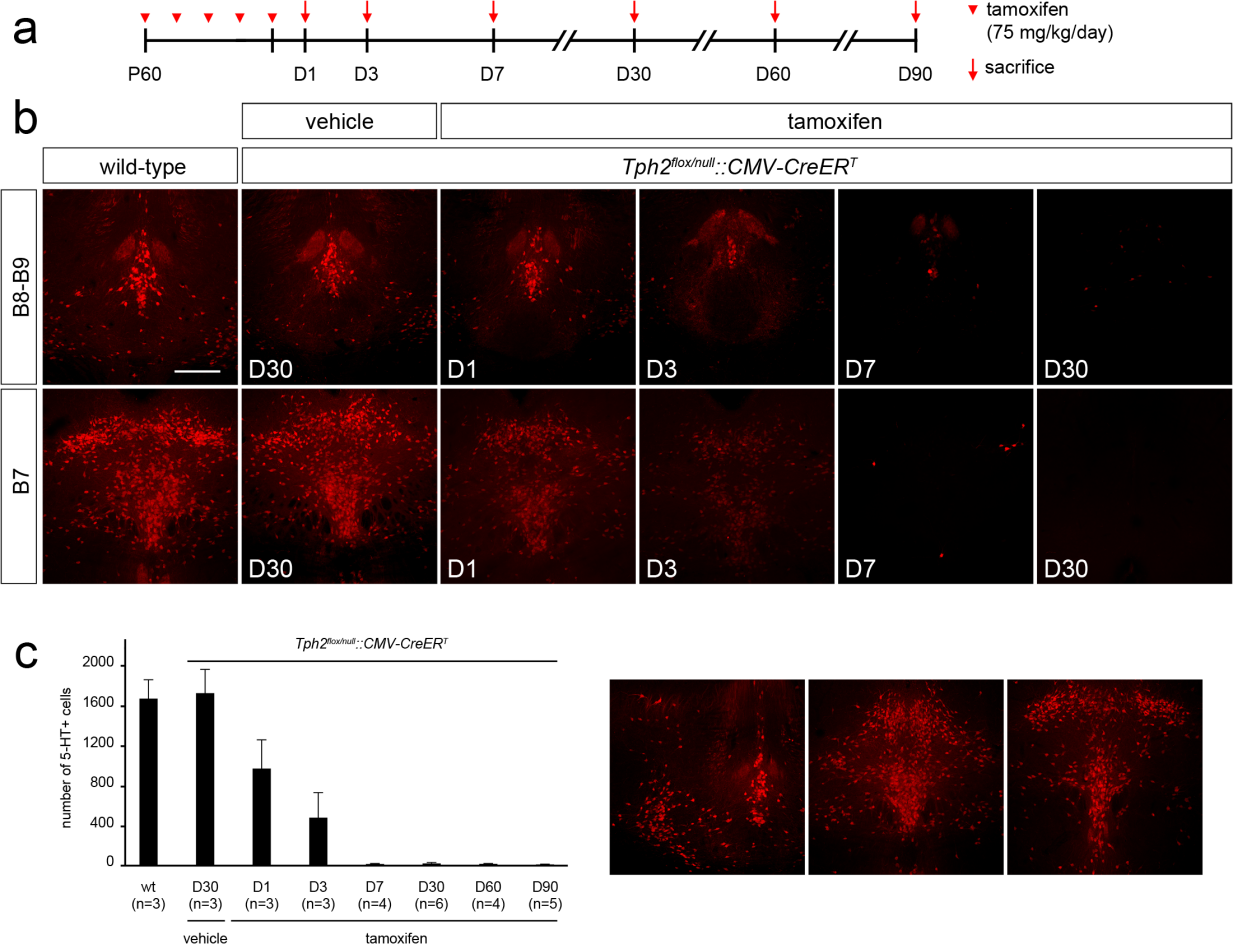
Tamoxifen-induced somatic recombination results in a rapid depletion of brain serotonin in adult mice. (**a**) Experimental design: *Tph2* ^*flox/null*^::*CMV-CreER* ^*T*^ mice received tamoxifen injection once per day starting from P60 for 5 consecutive days and sacrificed 1 (D1), 3 (D3), 7 (D7), 30 (D30), 60 (D60) or 90 (D90) days after the end of the treatment, respectively. (**b**) Representative coronal section of B8-B9 and B7 *raphe* nuclei showing serotonin immunoreactivity in (from right to left) adult wild-type mice, vehicle-treated *Tph2* ^*flox/null*^::*CMV-CreER* ^*T*^ control mice sacrificed 30 days after the last injection, tamoxifen-treated *Tph2* ^*flox/null*^::*CMV-CreER* ^*T*^ mice sacrificed 1, 3, 7 and 30 days after the end of treatment, respectively. (**c**) Histogram showing the number of serotonin immunoreactive cells in tamoxifen-treated *Tph2* ^*flox/null*^::*CMV-CreER* ^*T*^ mice, as compared to wild-type and vehicle-treated *Tph2* ^*flox/null*^::*CMV-CreER* ^*T*^ control mice. On the right, three representative coronal sections of B8-B9 and B7 *raphe* nuclei showing the distinct levels where serotonin positive neurons were counted. While the number of serotonin immunoreactive cells is unchanged between vehicle-treated *Tph2* ^*flox/null*^::*CMV-CreER* ^*T*^ and wild-type animals, a progressive and rapid reduction of serotonin immunoreactive neurons is observed after tamoxifen treatment, resulting in brain serotonin depletion starting from 7 days after the end of the treatment. Data are presented as mean ± SEM. Scale bar: 400 μm.

We next wanted to test whether the *Tph2* ^*flox/flox*^ allele could be effectively used for generating somatic *Tph2* null mutations to deplete serotonin content in a time- and tissue-controlled manner. First, we mated *Tph2* ^*flox/flox*^ mice to *Tph2* ^*null/null*^ mice to obtain trans-heterozygous *Tph2* ^*flox/null*^ mice, in which somatic recombination is facilitated thanks to the presence of a single floxed allele [64]. Then, *Tph2* ^*flox/null*^ mice were intercrossed with the *CMV-CreER* ^*T*^ mouse line, in which *Cre* recombinase is driven by an ubiquitous promoter and it is conditionally activated upon administration of tamoxifen (TM; [55]), in order to obtain *Tph2* ^*flox/null*^::*CMV*-*CreER* ^*T*^ mice. We evaluated the efficiency of tamoxifen-induced recombination of the *Tph2* third exon by monitoring the levels of serotonin immunoreactivity in the *raphe* of TM-treated *Tph2* ^*flox/null*^::*CMV-CreER* ^*T*^ animals. The dose and conditions were optimized on the basis of the maximal degree of Cre-ER^T^-induced loss of enzymatic activity of Tph2, as assessed by anti-serotonin immunostaining. Tamoxifen was administered to mice starting from P60 for five consecutive days and the effect of the treatment on serotonin content in the *raphe* nuclei was evaluated after 1 day (D1), 3 days (D3), 7 days (D7) and 30 days (D30) after the last injection (Fig 5A). Results showed a progressive reduction of immunofluorescence intensity already at D1, that gradually faded after D3 (Fig 5B), allowing the detection of only very few scattered immunoreactive neurons at D7, still showing apparently normal levels of serotonin (Fig 5B). The number of serotonin-immunoreactive neurons in TM-treated *Tph2* ^*flox/null*^::*CMV-CreER* ^*T*^ mice did not further decrease between D7 and D30, indicating that tamoxifen treatment had reached a peak of somatic recombination efficiency already one week after the last injection (Fig 5B and 5C). In contrast, serotonin immunoreactivity in the *raphe* nuclei of *Tph2* ^*flox/null*^::*CMV-CreER* ^*T*^ animals treated with vehicle as control resulted undistinguishable from that of wild-type or *Tph2* ^*flox/null*^ mice (Fig 5B). These data demonstrated that tamoxifen treatment could efficiently abrogate serotonin synthesis upon Cre-mediated conditional inactivation of *Tph2* expression.

Taken together, our results showed that Cre-mediated excision of the third exon of *Tph2* gene resulted in the depletion of serotonin content in the brain, which in turn elicited the features of serotonin-depleted models. Importantly, we demonstrated that serotonin synthesis could be conditionally ablated in the *Tph2* floxed mice, thus providing a valuable genetic tool to study the impact of serotonin homeostasis perturbation in a time-controlled manner.

## Discussion

Serotonin has been gaining increasing interest in the last decades for its function as neurotransmitter in central nervous system and as growth regulator during development. The dual role of this monoamine is relevant for the hypothesis that dysfunction of 5-HT homeostasis during critical periods of brain plasticity during development can be at the origin of a number of human pathologies with a known neurodevelopmental contribution, such as autism and mental retardation [16,39,41,44,46]. Moreover, non-physiological fluctuation of serotonin levels in adults might be associated with neuropsychiatric disorders such as depression and anxiety [40,42,45]. Consistently, the main current treatment of affective disorders is based on pharmacological approaches that target serotonin neurotransmission, such as selective serotonin reuptake inhibitors (SSRIs).

Hence, mouse genetic models allowing the study of the consequences of serotonin depletion in a time-specific manner represent valuable tools to identify the molecular basis of the aetiology of such disorders.

In the present study, we used a genetic approach based on homologous recombination in mouse ES cells to introduce *loxP* sites flanking the third exon of *Tph2* gene, in order to achieve a time-conditionally depletion of brain serotonin. In particular, we showed that the presence of the *loxP* sites in the *Tph2* genetic *locus* of *Tph2* ^*flox/flox*^ mice does not result in any obvious change in the synthesis of brain serotonin, meaning that the regulation of *Tph2* gene is maintained at physiological levels. Conversely, the excision of the third exon of *Tph2* is sufficient to induce depletion of serotonin in the brain and to promote those phenotypic characteristics typical of *Tph2* conventional knockouts, such as growth retardation and postnatal mortality [9,24]. Consistently with what observed in *Tph2* knockouts [9,24,26], reviewed in [44], or in other animal models lacking genes important for the phenotypic specification of serotonergic neurons, as *Pet1* [65] and *Lmx1b* [66] knockouts mice, no gross brain abnormalities have been shown in our *Tph2* depleted mice (*Tph2* ^*null/null*^).

Interestingly, our RT-PCR results indicated that in the *Tph2* ^*null*^ allele a second splicing variant, in which both the third and fourth *Tph2* exon are lacking (*Tph2Δ3Δ4*), is present together with the expected Tph2Δ3 transcript. The *Tph2Δ3Δ4* isoform is not physiologically present in the mouse brain, as it was undetectable in wild-type mice. Despite neither *Tph2Δ3* nor *Tph2Δ3Δ4* isoforms codified for a fully functional Tph2 protein, as shown by the dramatic depletion of serotonin immunodetection, a deeper analysis using higher sensitive detection methods did show a small remnant of serotonin in the *raphe* nuclei of *Tph2* ^*null/null*^ mice. From our data, it is likely that the observed residual serotonin could be explained by an enzymatic activity retained by the protein coded by the *Tph2Δ3Δ4* splicing variant, rather than by a Tph1-mediated synthesis, consistent with the absence of a Tph1 compensation in *Tph2* knockouts (reviewed in [44] and references therein).

The lack of the third exon of *Tph2* in *Tph2* ^*null/null*^ mice produces a frameshift, unmasking a stop codon within the fourth exon and producing a truncated enzyme. On the contrary, the correct reading frame is restored in the *Tph2Δ3Δ4* isoform, producing an enzyme with an internal deletion of residues 84–178, potentially retaining limited enzymatic activity. Yang and collaborators described a similar situation occurring in a published conditional knockout line for *Fntb*, the gene encoding the β-subunit of FTase [67]. These authors observed in the mutant allele that the mRNA splicing machinery produced a transcript coding for a protein with a short in-frame deletion, which explained the unexpected phenotypic results observed in the generated conditional knockout line [67,68].

Based on the aim of our study and due to the lack of comprehensive information about Tph2 structure, it is not possible to undoubtedly determine whether *Tph2Δ3Δ4*-derived protein can retain enzymatic activity. In fact, structural and biochemical information about the role of the three functional domains (i.e. regulatory, catalytic and tetramerization) in protein solubility, stability and activity have only beginning to be gathered. Several lines of evidences have shown that not only the catalytic domain, but also the N- and C-terminal domains, responsible for enzymatic regulation and tetramerization, respectively, have an important role in the enzymatic activity of both Tph1 and Tph2, which is also conserved among species [69–72]. In particular, deletion of the entire regulatory N-terminus alone or in combination with the concurrent removal of 24 aminoacids at the C-terminus has shown to profoundly affect human TPH2 biochemical properties, and pointed to the N-terminal region of the protein as the responsible of its poor solubility and stability [69]. Moreover, studies on human TPH2 missense variants have shown that mutations within the catalytic or the tetramerization domain affect the enzymatic activity more profoundly than those occurring at the N-terminal domain, and identified important residues that may be responsible of protein stability and, indirectly, of its activity [73]. *Tph2Δ3Δ4* isoform likely results in the production of an enzyme with a short internal truncation corresponding to residues 84–150, belonging to the regulatory N-terminus, and 151–178, located in the catalytic domain. To our knowledge, however, biochemical studies on internally deleted *Tph2* mutants are still missing and the described *Tph2Δ3Δ4* isoform represents the first internal deletion example preserving potential hydroxylase activity *in vivo*. Future *in vitro* studies and further characterization of the *Tph2Δ3Δ4* isoform will be needed to relate biochemical features with *in vivo* data to define the biochemical properties and structural changes of the mutant *Tph2* and to understand how the Δ3Δ4 deletion can promote such a drop in its activity.

Importantly, our results have also shown that the *Tph2* ^*flox/flox*^ mouse line can be efficiently used in combination with inducible Cre-expressing mouse line to effectively reduce serotonin levels in the brain of adult animals. Temporal analysis showed that depletion of serotonin occurred quickly and progressively in tamoxifen-treated *Tph2* ^*null/flox*^::*CMV- CreER* ^*T*^ mice with only few serotonergic neurons immunoreactive for serotonin still present after 7 days (D7) after tamoxifen treatment and dropping to less than 1% at D30. As compared to the *Tph2* conditional mouse line generated by Kriegebaum and collaborators [51], our floxed allele appears to be more suitable for time-inducible serotonin synthesis inactivation when combined to inducible Cre-expressing mouse lines. In fact, despite a similar tamoxifen administration protocol was used with the two distinct lines, our *Tph2* floxed allele showed higher effectiveness as Cre-mediated somatic recombination occurred in nearly all serotonergic neurons. Such a difference in recombination efficiency may be ascribed to at least two factors. On one side, the shorter distance between the two *loxP* sites in our allele (i.e. 0.57 Kb as compared to the 3.4 Kb), as it is known that distance between *loxP* sites negatively affects the recombination efficiency [74,75]. On the other, in our study we used trans-heterozygous *Tph2* ^*flox/null*^ mice in which somatic recombination is likely facilitated thanks to the presence of a single floxed allele [64].

On the whole, our study showed that the excision of the third exon of *Tph2* gene is sufficient to drastically reduce the amount of serotonin content in the brain, and provided evidence that an alternative splicing event induced by the recombination may account for the remnant of serotonin in the *raphe*, which nevertheless did not prevent *Tph2* ^*null/null*^ mice from displaying *Tph2* conventional knockout-like features. In the future, the use of the *Tph2* ^*flox/flox*^ line will open the possibility to investigate the consequences of time-specific serotonin depletion on brain functioning and behaviour, as well as on serotonergic system itself, in order to evaluate the sensitivity of the mature serotonergic circuitry to non-physiological serotonin fluctuation.

## Supporting Information

S1 Fig *Tph2* ^*null/null*^ adult mice are depleted of brain serotonin but show a remnant of 5-HT in few scattered serotonergic neurons. Low (**a**-**c**, **d**-**f**) and high (**c’**, **c”**, **f**’, **f”**) magnification confocal images showing serotonin immunoreactivity on coronal section of B8-B9 (**a**-**c”**) and B7 (**d**-**f”**) *raphe* nuclei of wild-type (**a**, **d**), *Tph2* ^*flox/flox*^ (**b**, **e**), *Tph2* ^*null/null*^ (**c**, **c’**, **f**, **f’**) and *Tph2*::*eGFP*-/- (**c”**, **f”**) adult mice. Boxes in **c**, **f** highlight the region of *raphe* shown at high magnification in **c’**, **f’**. Immunofluorescence signalling was increased through the use of artificial gain in high magnification images (**c’**-**c”**, **f**’-**f”**). Serotonin immunoreactive neurons in *Tph2* ^*flox/flox*^ adult animals are present in comparable number and distribution as in wild-type controls (**a-b**, **d-e**), while brain serotonin depletion is evident in *Tph2* ^*null/null*^ mice (**c**, **f**). Artificial gain of high magnification images allows to detect the presence of a remnant of serotonin in few serotonergic neurons in both B8-B9 and B7 *raphe* nuclei of *Tph2* ^*null/null*^ brains (**c’**, **f’**). The use of the same parameters for image acquisition and data processing on *Tph2*::*eGFP*-/- *raphe* sections immunostained for serotonin failed to show any detectable immunoreactivity for serotonin (**c”**, **f”**). Scale bar: (**a-c**, **d-f**) 400 μm, (**c’**, **c”, f’, f”**) 100 μm.
(TIF)

S2 Fig *Tph1* and *Tph2* expression in *Tph2* ^*null/null*^ brain, liver and pineal gland. (**a**) Low magnification image of a *Tph2* ^*null/null*^ mouse coronal brain section hybridized with a ^35^S-labelled antisense riboprobe specific for *Tph1* and counterstained with Giemsa. The pineal gland and both dorsal and median *raphe* nuclei are present in the section shown. (**b**) X-ray film autoradiography showing the mouse coronal brain section present in **a** confirming *Tph1* expression in the in the pineal gland but not in the *raphe* nuclei. (**c**) Agarose gel electrophoresis analysis of RT-PCR experiments showing the expression of *Tph1*, *Tph2* and *β-actin* in the liver, brain and pineal gland of *Tph2* ^*null/null*^ mice. Results demonstrate that *Tph1* expression is present in the pineal gland, whereas *Tph2* expression is detectable in the brain but absent in liver and pineal gland. In the lower panel is reported the expression of *β-actin* used as a positive control. P: pineal gland; DR: dorsal *raphe* nucleus; MnR: median *raphe* nucleus; I: ladder; L: liver; B: brain; rt-: no reverse transcription. Scale bar: 750 μm.
(TIF)
