# Supplementary figures and images for: Generation of a Tph2 Conditional Knockout Mouse Line for Time- and Tissue-Specific Depletion of Brain Serotonin
Source: PLoS One. 2015 Aug 20;10(8):e0136422. doi: 10.1371/journal.pone.0136422 (PMC4546246; doi:10.1371/journal.pone.0136422)

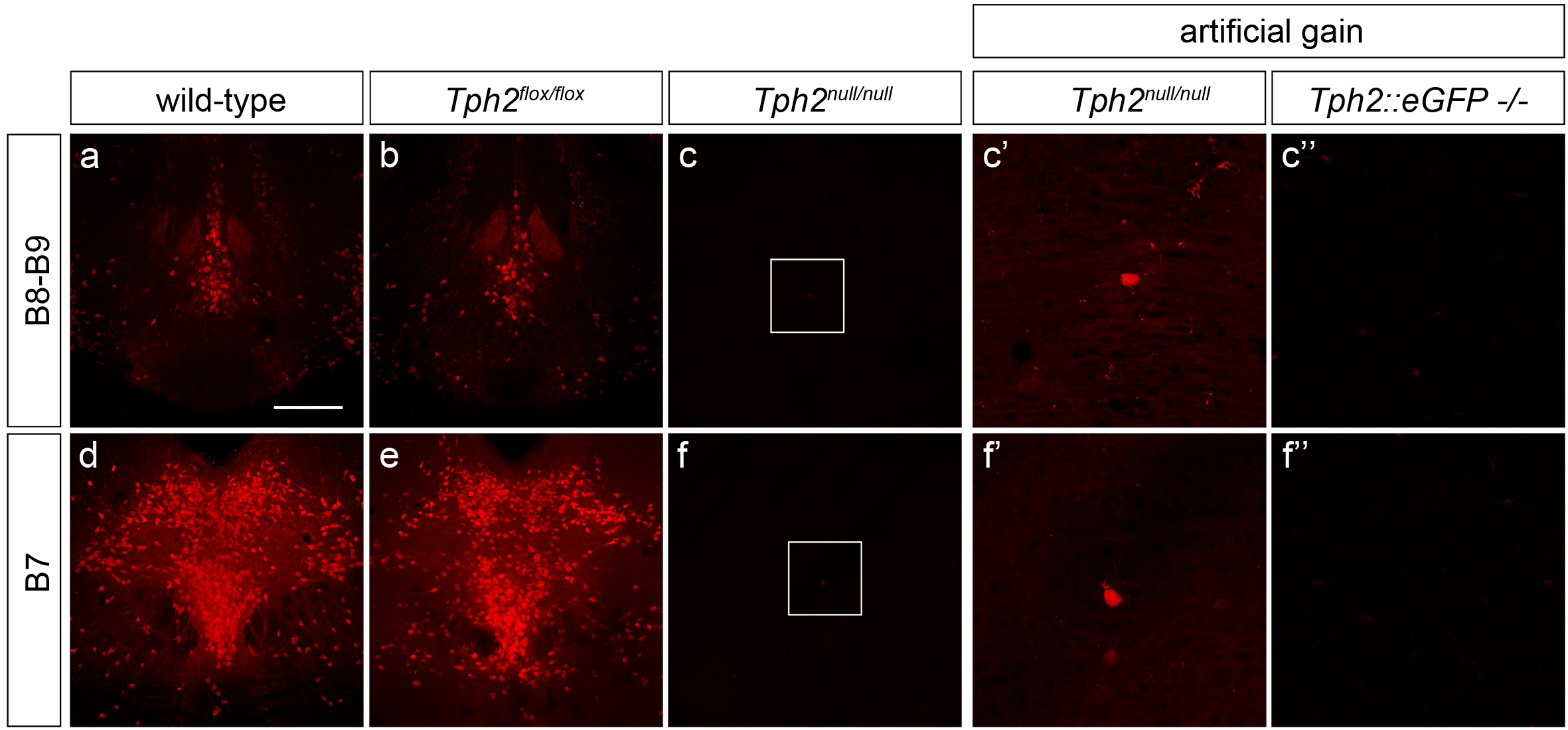

Supplement: S1 Fig — Low (a-c, d-f) and high (c’, c”, f’, f”) magnification confocal images showing serotonin immunoreactivity on coronal section of B8-B9 (a-c”) and B7 (d-f”) raphe nuclei of wild-type (a, d), Tph2 flox/flox (b, e), Tph2 null/null (c, c’, f, f’) and Tph2::eGFP-/- (c”, f”) adult mice. Boxes in c, f highlight the region of raphe shown at high magnification in c’, f’. Immunofluorescence signalling was increased through the use of artificial gain in high magnification images (c’-c”, f’-f”). Serotonin immunoreactive neurons in Tph2 flox/flox adult animals are present in comparable number and distribution as in wild-type controls (a-b, d-e), while brain serotonin depletion is evident in Tph2 null/null mice (c, f). Artificial gain of high magnification images allows to detect the presence of a remnant of serotonin in few serotonergic neurons in both B8-B9 and B7 raphe nuclei of Tph2 null/null brains (c’, f’). The use of the same parameters for image acquisition and data processing on Tph2::eGFP-/- raphe sections immunostained for serotonin failed to show any detectable immunoreactivity for serotonin (c”, f”). Scale bar: (a-c, d-f) 400 μm, (c’, c”, f’, f”) 100 μm. (TIF) [file pone.0136422.s001.tif]

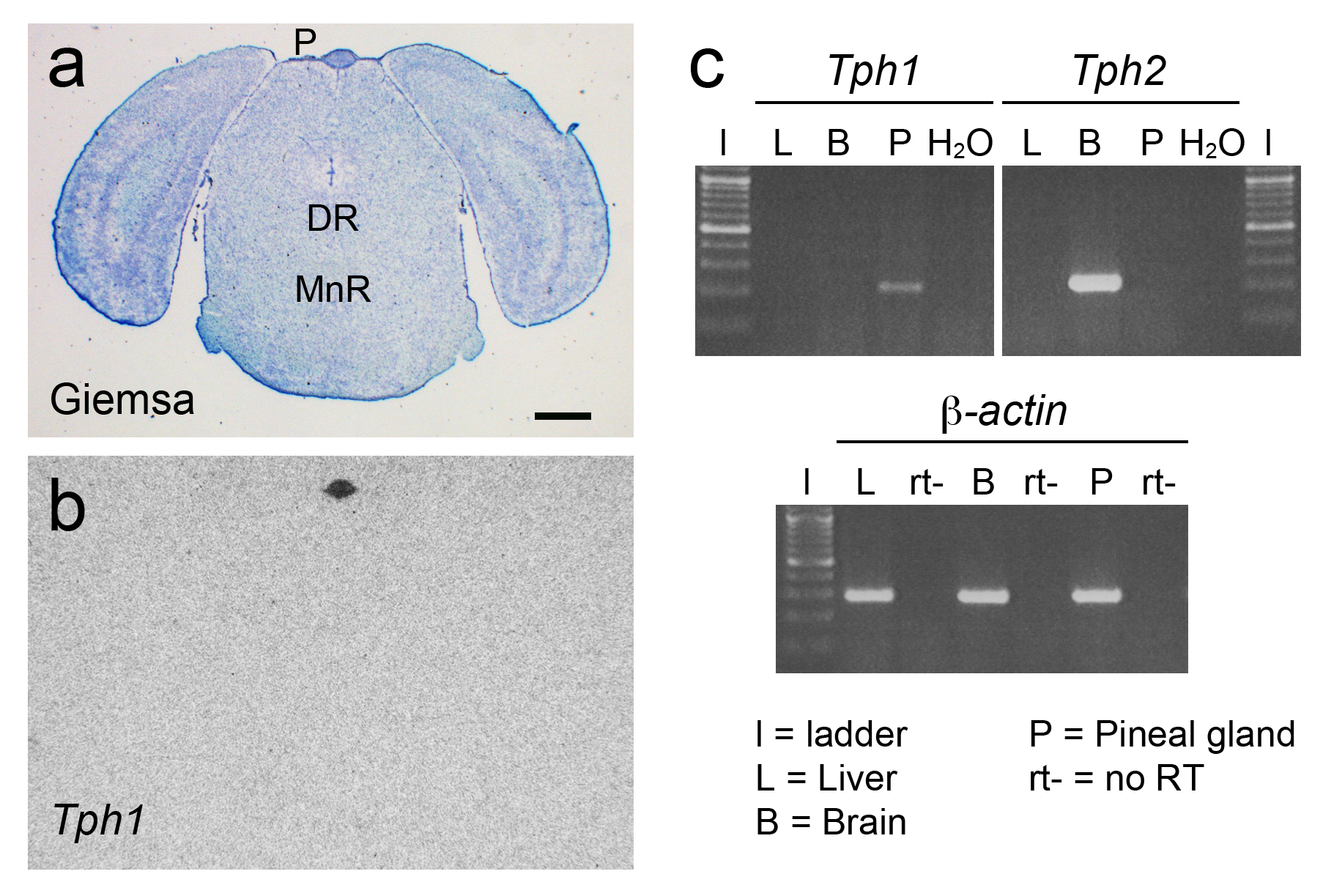

Supplement: S2 Fig — (a) Low magnification image of a Tph2 null/null mouse coronal brain section hybridized with a 35S-labelled antisense riboprobe specific for Tph1 and counterstained with Giemsa. The pineal gland and both dorsal and median raphe nuclei are present in the section shown. (b) X-ray film autoradiography showing the mouse coronal brain section present in a confirming Tph1 expression in the in the pineal gland but not in the raphe nuclei. (c) Agarose gel electrophoresis analysis of RT-PCR experiments showing the expression of Tph1, Tph2 and β-actin in the liver, brain and pineal gland of Tph2 null/null mice. Results demonstrate that Tph1 expression is present in the pineal gland, whereas Tph2 expression is detectable in the brain but absent in liver and pineal gland. In the lower panel is reported the expression of β-actin used as a positive control. P: pineal gland; DR: dorsal raphe nucleus; MnR: median raphe nucleus; I: ladder; L: liver; B: brain; rt-: no reverse transcription. Scale bar: 750 μm. (TIF) [file pone.0136422.s002.tif]
